# Negative Appendicectomy Rate and Surgery Despite Negative Triple-Screen Inflammatory Markers in Children: A Single-Centre Retrospective Cohort Study

**DOI:** 10.7759/cureus.117864

**Published:** 2026-10-13

**Authors:** Neal Pun, Sultan Aydemir

**Affiliations:** 1 General Surgery, Queens Hospital, University Hospitals of Derby and Burton NHS Foundation Trust, Burton, GBR; 2 General Surgery, University Hospitals of Derby and Burton NHS Foundation Trust, Burton, GBR

**Keywords:** academy of medical royal colleges, diagnostic accuracy, girft (getting it right first time), negative appendectomy rate, paediatric appendicitis, paediatric appendicitis score, paediatric surgery, quality improvement research, retrospective cohort study, triple-screen inflammatory markers

## Abstract

Introduction

The negative appendicectomy rate (NAR) is an important measure of diagnostic accuracy and quality of care. Diagnosing appendicitis in children remains challenging due to frequently atypical and non-specific clinical presentations. Despite advances in clinical scoring and imaging, NAR in the UK remains high. The primary aims of this audit were to determine the NAR at Queen's Hospital Burton against the Getting It Right First Time (GIRFT)-reported national figure, and to establish how often surgery proceeded despite negative Academy of Medical Royal Colleges (AoMRC) triple-screen inflammatory markers, to inform review of the local diagnostic pathway. Exploratory analyses examined the association of Paediatric Appendicitis Score (PAS) risk category and preoperative imaging with NAR.

Methods

This retrospective, single-centre, single-cycle audit included 318 children aged 5-16 years who underwent surgical exploration for suspected acute appendicitis between December 2017 and June 2026; 302 had appendicectomy with available histopathology and were included in the primary analysis. Data were retrospectively collected from electronic patient records, including demographics, inflammatory markers, imaging, histopathology, and post-operative complications. Negative appendicectomy was defined histologically as the absence of inflammatory changes. Triple-screen-negative was defined as CRP <10 mg/L, WCC <10.5 ×10⁹/L, and neutrophil percentage <75% on the first pre-operative sample. As secondary, exploratory analyses, the PAS was reconstructed retrospectively, blinded to histopathology, and the association of PAS risk category and preoperative imaging with NAR was examined. Statistical analysis used Microsoft Excel for the web (Microsoft Corp., Redmond, WA), with Fisher's exact test and ROC/AUC analyses performed in GraphPad (Dotmatics, Boston, MA) and jamovi (https://www.jamovi.org), respectively.

Results

Of 302 children who underwent appendicectomy with available histopathology, the NAR was 21.2% (95% CI 17.0-26.1%), significantly higher than the GIRFT-reported national figure of 10% (two-sided exact binomial test, p<0.0001). Of 318 children explored, 55 (17.3%) met AoMRC triple-screen-negative criteria yet proceeded to surgery; among the 49 with histopathology, the NAR was 73.5% (36/49; 95% CI 59.7-83.8%). In exploratory analyses, NAR fell across PAS risk categories, from 50.0% (low risk) to 31.1% (equivocal risk) to 10.0% (high risk) (χ² p≈1.65×10⁻⁶), and PAS had an AUC of 0.730 (95% CI 0.663-0.798) for appendicitis. Imaging use declined from low risk (52.6%) to high risk (27.6%). Within the equivocal-risk group, NAR was higher among imaged than non-imaged patients (48.9%, 23/47 vs 21.2%, 18/85; OR 3.57, 95% CI 1.65-7.73; Fisher's exact p=0.0015).

Conclusion

NAR at this centre significantly exceeded the GIRFT-reported national figure. A substantial minority of children who underwent surgery (17.3%) met AoMRC triple-screen-negative criteria, and the NAR in this subgroup was high. These findings identify an opportunity to review the local diagnostic pathway. Prospective evaluation is required to determine whether standardised, combined use of a validated clinical scoring system (PAS), biochemical inflammatory markers, and imaging can reduce unnecessary appendicectomy without increasing missed or delayed diagnoses.

## Introduction

Acute appendicitis is one of the most common surgical emergencies among the paediatric population, and though prior literature has demonstrated low mortality [1], removing a normal appendix exposes children to general anaesthetic, avoidable operative and post-operative risks, hospital stay, and healthcare costs. The negative appendicectomy rate (NAR) is therefore an important measure of diagnostic accuracy and quality of care [1,2]. 

Clinical assessment, inflammatory markers, and imaging are commonly used together when assessing suspected appendicitis. In children, combining inflammatory markers such as white cell count (WCC), neutrophils, and C-reactive protein (CRP) has been shown to have a high negative predictive value (NPV). One paediatric study found that among children with all three inflammatory markers negative who underwent appendicectomy, 89.5% had a normal appendix on histology [3]. Reassuring diagnostic imaging, including ultrasound and computed tomography (CT), has also been associated with lower negative appendicectomy rates in children [4]. 

The concept of ‘triple-screen-negative’ in this audit focuses on the clinically reassuring biochemical inflammatory markers. The Academy of Medical Royal Colleges (AoMRC) has stated that a negative triple-screen - CRP <10 mg/L, WCC <10.5 ×10⁹/L, and neutrophil percentage <75% - carries an NPV >99% for excluding appendicitis, with imaging not recommended in this setting [5]. This guidance states that it applies to adults and children; however, the NPV figure itself appears within a discussion of adult assessment, with no paediatric-specific evidence cited in support. A comparably high combined NPV (98.7%) for the same three markers has been reported in a large paediatric cohort, though using cut-offs not confirmed to be identical to AoMRC's [3]. 

Currently, there is limited paediatric-specific evidence examining how often children meeting this guidance proceed to appendicectomy, and with what outcome. Most existing studies assess individual biochemical inflammatory markers, or combine clinical scores, blood tests, and imaging, rather than focusing specifically on triple-screen-negative inflammatory markers as a defined threshold [3-5].

This audit reviewed an 8.5-year cohort of children aged 5-16 years undergoing surgical exploration for suspected appendicitis between December 2017 and June 2026, selected to ensure an adequate sample size given the single-centre setting. The primary aims were to determine the negative appendicectomy rate against the national NAR reported by Getting It Right First Time (GIRFT) [6], and to identify the proportion and characteristics of AoMRC triple-screen-negative cases [5] proceeding to surgery. As a secondary, exploratory aim, the audit examined the diagnostic performance of the Paediatric Appendicitis Score (PAS) when applied retrospectively and described patterns of imaging use in relation to PAS risk category and negative appendicectomy rate. 

## Materials and methods

Audit approval 

This audit received local audit approval from Queen’s Hospital Burton. Queen’s Hospital Burton serves as a secondary care site for emergency surgical procedures. 

Audit design 

This retrospective, single-centre, single-cycle audit included children undergoing surgical exploration for suspected acute appendicitis; those with appendicectomy and available histopathology were included in the primary analysis. The audit tested the local practice against two external standards: the national NAR of 10% reported by GIRFT [6] and the AoMRC triple-screen guidance [5]. Diagnostic performance of the PAS and patterns of preoperative imaging use are reported as secondary, exploratory analyses supporting the audit's recommendations, since PAS was not used prospectively and imaging use was not systematically protocolised during the study period. 

No individual patient-level data are reported. All results were reported as aggregated total frequencies, percentages, and summary diagnostic accuracy statistics (including area under the curve (AUC)). 

Setting and population 

The audit included all patients aged 5-16 years who underwent surgical exploration for suspected acute appendicitis between December 2017 and June 2026, to allow adequate sample size. Cases were identified retrospectively via monthly Electronic Patient Records (EPR) searches, using the paediatric ward code, for all admissions under the General Surgery team; each admission was then manually reviewed against admission documentation, operative notes, and histopathology to confirm suspected appendicitis and determine whether appendicectomy had been performed. This admission-based approach, rather than a procedure-specific code, meant diagnostic laparoscopies with a normal appendix left in situ were captured alongside appendicectomies. As searches covered every month of the study period, ascertainment was consecutive, and as no child underwent surgical exploration more than once during this time, each of the 318 episodes represents a distinct patient. 

Inclusion criteria 

Patients aged 5-16 years presenting with suspected acute appendicitis who underwent surgical exploration were included.

Exclusion criteria 

Patients were excluded if they had undergone interval or incidental appendicectomy during unrelated surgery, had known chronic abdominal pathology unrelated to the presenting suspected appendicitis, or had missing critical data that precluded meaningful analysis. No patient met these criteria. Children transferred to a paediatric tertiary specialist centre without surgical exploration at Queen's Hospital Burton were not eligible. Patients who were explored but had a macroscopically normal appendix left in situ, and therefore no histopathology, remained in the audit cohort but were excluded from the NAR and diagnostic accuracy denominators. Patients with insufficient documentation to calculate a PAS were excluded from PAS-related analyses only. 

Data collection 

Data were retrospectively collected from the EPR system using a standardised proforma, incorporating information from clinical notes, operative records, pathology reports, and discharge summaries. Data extraction was performed independently by two reviewers, with discrepancies resolved by consensus, to minimise inter-observer variability in retrospective data collection and PAS calculation. All data were de-identified and securely stored on a protected workbook with restricted access. Collected variables included demographic data (age and sex), inflammatory markers (white cell count, neutrophil count, and C-reactive protein), temperature, and the PAS, calculated from documented clinical and laboratory findings. Imaging modality and reported conclusion, operative findings, histopathology results, and post-operative complications (surgical site infection (SSI), recurrent abdominal pain, post-operative ileus, and intra-abdominal collection) were also recorded. Post-operative complications were ascertained through re-presentation to Queen's Hospital Burton within 30 days of surgery, identified via EPR review; complications managed at other hospitals could not be captured. Each affected child had a single complication type recorded, with no instances of overlapping complications within individual patients. Histopathology results were subsequently categorised into normal appendix with no inflammation, uncomplicated appendicitis, and complicated appendicitis. 

PAS was not formally documented in clinical notes during the study period. Scores were calculated retrospectively, for the purpose of this audit, for all patients with sufficient documentation based on clinical and laboratory findings in the medical notes. As such, retrospective PAS could not have influenced the original clinical decision to proceed to surgery; its diagnostic performance is reported to evaluate its potential utility if prospectively implemented, consistent with GIRFT's recommendation that a validated scoring system be embedded in the diagnostic pathway for suspected acute appendicitis [7]. Cases with insufficient documentation to calculate PAS (n=4) were identified and excluded from PAS-related analyses. 

PAS completeness was assessed using the same criteria regardless of histopathology availability; all four exclusions for insufficient PAS documentation occurred among patients with definitive histopathology, and all patients without histopathology had sufficient documentation to calculate PAS. These four exclusions reflected a missing temperature or blood test result - one of PAS's three objective, measured components - rather than a missing clinical symptom; the assume-absent convention described below applied only to the five clinical/examination symptom components, not to the objective, measured components, which required an actual recorded value and could not be assumed.

PAS components were extracted from emergency department, surgical, and nursing triage documentation recorded on the day of presentation; a symptom component was scored as present if documented in any of these sources, and as absent only when not recorded in any of the three, since clinical notes more reliably document positive than negative findings. No instances of direct contradiction between sources were identified. PAS was reconstructed before histopathology results were reviewed, so scoring was performed without knowledge of the eventual outcome. As with other data extraction in this audit, PAS components were independently assessed by two reviewers, with discrepancies resolved by consensus; a formal inter-rater agreement statistic was not calculated specifically for PAS reconstruction.

Definitions 

Negative appendicectomy was defined as removal of a histologically normal (non-inflamed) appendix, without significant inflammatory changes, confirmed by histopathology as the reference standard in this audit [8]. Histopathology was not available at the time of the decision to operate; a normal appendix is established only retrospectively. Triple-screen-negative was defined as CRP <10 mg/L, WCC <10.5 ×10⁹/L, and neutrophil percentage <75%, all measured pre-operatively [5]. Classification used the first (admission) pre-operative blood sample. Neutrophil percentage was calculated from the recorded absolute neutrophil count and white cell count. Complicated appendicitis was defined as histopathology showing gangrenous, necrotising, or perforated appendicitis with or without peritonitis/abscess [9-13]. 

The Paediatric Appendicitis Score (PAS) was calculated retrospectively from clinical findings and laboratory results documented at presentation to hospital, using the components, weighting, and numerical thresholds described by Samuel [14] and used in a comparable form by Goldman et al. [15]. The score comprised eight components: migration of pain to the right lower quadrant (1 point), anorexia (1 point), nausea or vomiting (1 point), fever >38°C (1 point), right lower quadrant tenderness (2 points), right lower quadrant tenderness with coughing, percussion, or hopping (2 points), white cell count >10 × 10⁹/L (1 point), and absolute neutrophil count >7.5 × 10⁹/L (1 point). Scores ranged from 0 to 10. Patients were stratified as low risk (PAS <4), equivocal risk (PAS 4-6), or high risk (PAS >6), a stratification in common clinical use [16]. 

Cases with no histopathology were defined as those in which surgical exploration was performed, but the appendix was not removed (a macroscopically normal appendix left in situ) and therefore not sent for histological analysis. These were excluded from the NAR and diagnostic accuracy denominators and are reported separately.

Imaging conclusion was categorised as suggestive of appendicitis (appendix visualised with evidence of appendiceal inflammation), no appendicitis (appendix visualised without evidence of inflammation), or inconclusive (appendix not visualised, precluding comment on the presence or absence of appendicitis). All imaging was performed pre-operatively, before the decision to operate, and no child underwent more than one imaging examination. 

Statistical analysis 

All statistical analyses were performed using Microsoft Excel for the web (Microsoft Corp., Redmond, WA). Fisher's exact test, not available as a native function in Microsoft Excel, was calculated using GraphPad QuickCalcs (Dotmatics, Boston, MA). Receiver operating characteristic curve analysis was performed using jamovi (Version 28.2, https://www.jamovi.org), with the ClinicoPath module (clinical ROC analysis). Categorical variables were summarised as frequencies and percentages, with 95% confidence intervals (CI) calculated using the Wilson score method given several small subgroup sizes. Where relevant, continuous variables were analysed as means with standard deviation or medians with ranges.

Primary analysis 

The NAR was compared against the national figure of 10% reported in the GIRFT National Specialty Report of Paediatric General Surgery and Urology (2021) [6], using a one-sample, two-tailed exact binomial test. GIRFT defines negative appendicectomy as appendicectomy for suspected appendicitis subsequently found not to represent appendicitis on histological examination, consistent with the definition used in this audit; however, GIRFT's report does not state the number of patients or the specific age range underlying this figure, beyond confirming a paediatric population, and the underlying data were collected in 2017-2018. 

The proportion of explored patients meeting AoMRC’s triple-screen-negative criteria, and the NAR within this subgroup, were calculated as simple proportions with 95% confidence intervals, as above. 

Secondary exploratory analysis 

Associations between PAS risk category and histological outcome, and between PAS risk category and imaging use, were assessed using the chi-square test. The difference in NAR between imaged and non-imaged patients within the equivocal risk group was assessed using Fisher's exact test, given small cell counts. Receiver operating characteristic (ROC) curve analysis was performed to evaluate the diagnostic performance of PAS in predicting appendicitis, using 298 patients with a documented PAS score and definitive histopathology. Analysis was conducted in jamovi (Version 28.2), using the ClinicoPath module (clinical ROC analysis); the AUC and 95% confidence interval were calculated using DeLong's method. 

As these secondary analyses were exploratory and intended to describe patterns within the diagnostic pathway rather than test independent primary hypotheses, formal adjustment for multiple comparisons was not applied. A p-value of <0.05 was considered statistically significant throughout. 

## Results

A total of 318 paediatric patients underwent surgical exploration for suspected acute appendicitis. Of these, 16 had a macroscopically normal appendix that was left in situ and therefore had no histopathology; they were excluded from the NAR and diagnostic accuracy denominators, leaving 302 patients for the primary analysis (Figure 1).

**Figure 1:**
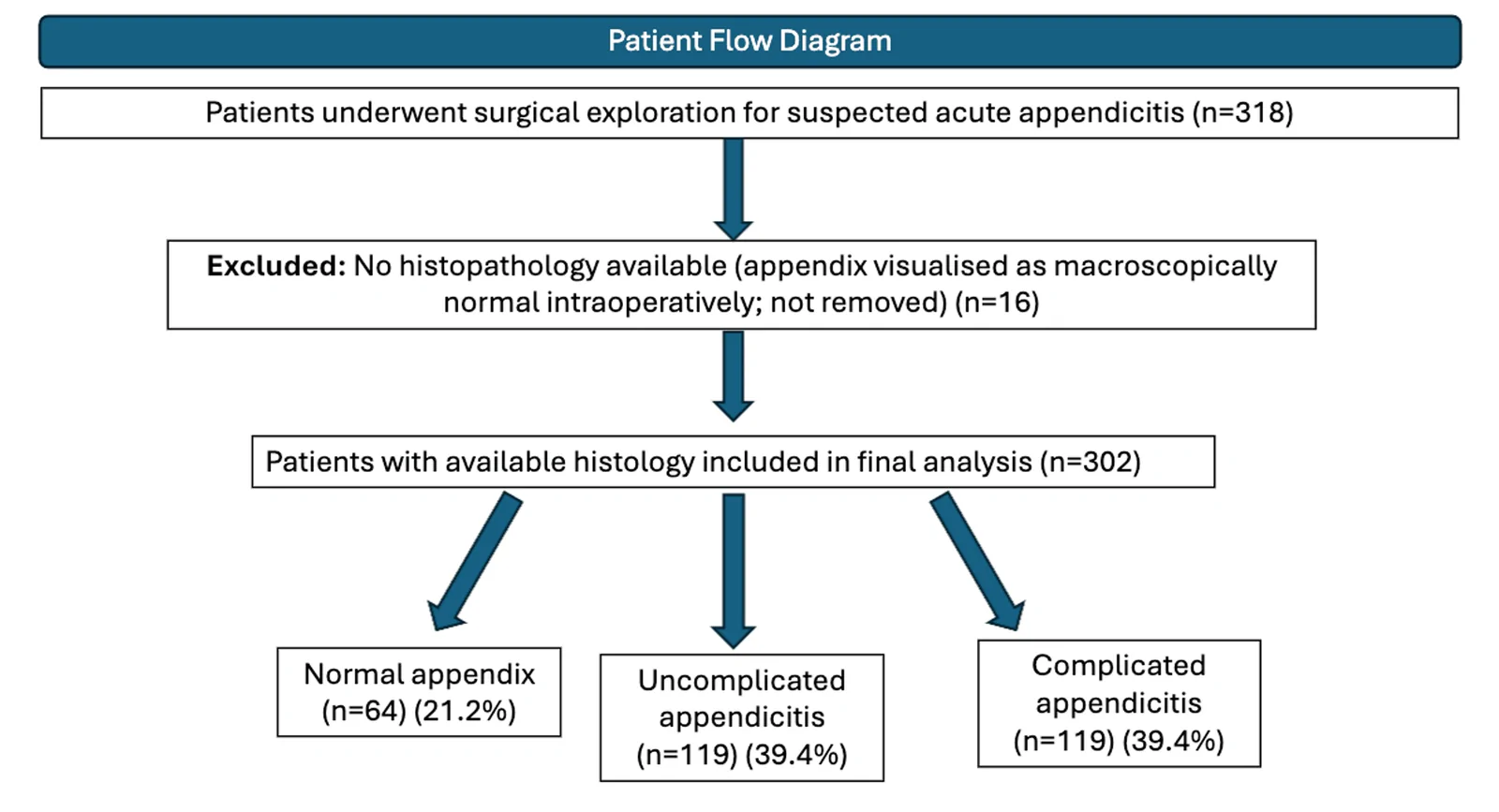
Patient Flow Diagram Of 318 patients who underwent surgical exploration for suspected acute appendicitis, 16 had a macroscopically normal appendix left in situ and no histopathology, leaving 302 for final analysis.​

One additional child was transferred to a paediatric tertiary specialist centre without surgical exploration at Queen's Hospital Burton and was not part of the cohort. Among the 302 patients, 64 (21.2%) had a normal appendix on histology, 119 (39.4%) had uncomplicated appendicitis, and 119 (39.4%) had complicated appendicitis. 

Patient demographics 

Clinical characteristics and baseline demographics are described in Table 1.

**Table 1:** Baseline Demographics​ Baseline demographic characteristics of the explored cohort (n=318), including age (mean ± standard deviation (SD)) and sex distribution.​

Characteristic ​	Value​
Total Explored (n)​	318​
Age, years (mean ± SD)​	11.66 ± 2.84​
Sex, n (%) - Male​	170 (53.5%)​
Sex, n (%) - Female​	148 (46.5%)​

The mean age was 11.66 ± 2.84 years, with the largest proportion of patients (43.7%) in the 12-15 years age band (Table 2).

**Table 2:** Age Band Distribution​ Distribution of patients across age bands within the operated cohort (n=318).

Age Band, years​	Value, n (%)​
5-8 ​	47 (14.8%)​
9-11​	105 (33.0%)​
12-15​	139 (43.7%)​
16 ​	27 (8.5%)​

In total, 170 patients (53.5%) were male and 148 (46.5%) were female (Figure 2).

**Figure 2:**
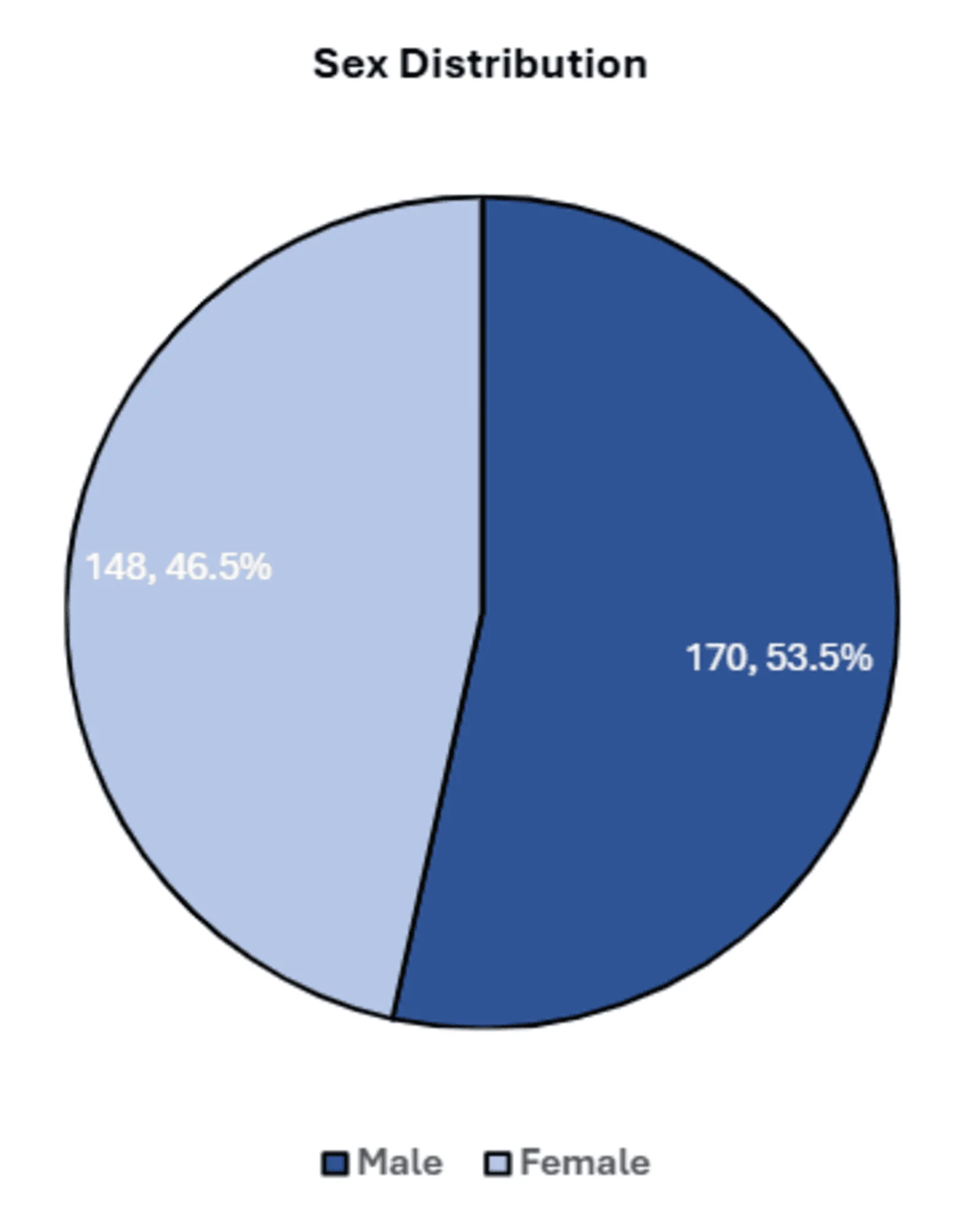
Sex Distribution Sex distribution of the operated cohort (n=318): 170 male (53.5%) and 148 female (46.5%).​

Negative appendicectomy rate vs GIRFT national NAR figure 

Of the 302 patients with histology, 64 had a negative appendicectomy, giving a NAR of 21.2% (95% CI 17.0-26.1%). A one-sample, two-tailed exact binomial test confirmed that this significantly exceeded the GIRFT-reported national NAR of 10% (2021) (p = 7.54 × 10⁻⁹). A total of 5 out of 64 (7.8%) patients were readmitted to hospital for post-operative complications; 3 (4.69%) with SSI and 2 (3.13%) with recurrent abdominal pain (Table 3).

**Table 3:** Post-Operative Complications - Negative Appendicectomy Group​ Post-operative complications requiring hospital readmission among patients with a negative appendicectomy (n=64).​

Complication​	Number (n)​	Percentage (of 64)​
Surgical site infection​	3​	4.69%​
Recurrent abdominal pain​	2​	3.13%​
Post-operative ileus​	0​	0%​
Intra-abdominal collection​	0​	0%​
Total​	5​	7.8%​

AoMRC triple-screen 

A total of 55 out of 318 patients who were operated on met the triple-screen negative AoMRC criteria, resulting in a proportion of 17.3% (95% CI 13.5-21.8%). Within the triple-screen negative cohort, 49/55 patients had available histology results. Histological breakdown further reveals that 36 (73.5%, 95% CI 59.7-83.8%) patients had a normal appendix, 12 patients (24.5%, 95% CI 14.6-38.1%) had uncomplicated appendicitis, and 1 patient (2.0%, 95% CI 0.4-10.7%) had complicated appendicitis (Table 4).

**Table 4:** Histology Breakdown - Triple-Screen-Negative Subgroup​ Histopathological outcomes among patients meeting the Academy of Medical Royal Colleges' (AoMRC) triple-screen-negative criteria with available histology (n=49).​

Histology outcome​	Number (n)​	Percentage (of 49)​
Normal appendix​	36​	73.5%​
Uncomplicated appendicitis​	12​	24.5%​
Complicated appendicitis​	1​	2.0%​

A total of 36 of the 64 patients (56.3%) with a negative appendectomy had already met AoMRC's triple-screen-negative criteria before surgery. NAR within the triple-screen-negative subgroup was therefore 73.5% (95% CI 59.7-83.8%). 7/55 patients in the operated cohort had post-operative complications needing readmission (regardless of whether histology results were available), including 4 (7.3%, 95% CI 2.9-17.3%) patients with SSI and 3 (5.5%, 95% CI 1.9-14.9%) patients with recurrent abdominal pain. 31/55 (56.4%, 95% CI 43.3-68.6%) of triple-screen-negative patients underwent imaging, despite AoMRC stating imaging is not recommended in this setting. Of these, 27 had definitive histology available, of whom 22 (81.5%, 95% CI 63.3-91.8%) had a normal appendix. Imaging conclusions were inconclusive in 24/31 (77.4%, 95% CI 60.2-88.6%), suggestive of appendicitis in 4/31 (12.9%, 95% CI 5.1-28.9%), and reported as no appendicitis in 3/31 (9.7%, 95% CI 3.3-24.9%). 

Secondary/exploratory analysis: PAS and imaging use 

Of the 302 patients with available histology, 298 had sufficient documentation to assign a PAS risk category and a corresponding numeric score and were included in both the diagnostic accuracy and ROC curve analyses; the remaining 4 had insufficient documentation to calculate PAS. Patients were categorised according to PAS score into low risk, equivocal risk, or high risk, per the thresholds defined in the Materials and Methods section. True positive (TP), false positive (FP), false negative (FN), and true negative (TN) were defined using PAS low risk as test-negative and equivocal or high risk as test-positive. TP was reported at 226, FP was reported at 56, FN was reported at 8, and TN was reported at 8 (Table 5).

**Table 5:** PAS 2×2 Diagnostic Accuracy Table​ 2×2 contingency table for Paediatric Appendicitis Score (PAS) diagnostic accuracy against histopathological outcome, using a positive-test threshold of equivocal or high risk (n=298). TP: true positive; FP: false positive; FN: false negative; TN: true negative.​

​Parameters	Appendicitis confirmed​	Normal appendix​	Total​
PAS positive (equivocal/high risk)​	TP = 226​	FP = 56​	282​
PAS negative (low risk)​	FN = 8​	TN = 8​	16​
Total​	234​	64​	298​

Specificity, sensitivity, positive predictive value (PPV), and NPV were also calculated, each with a 95% CI. Analysis showed sensitivity was 96.6% (95% CI 93.4-98.3%) and specificity was 12.5% (95% CI 6.5-22.8%). PPV was reported at 80.1% (95% CI 75.1-84.4%) and NPV at 50.0% (95% CI 28.0-72.0%) (Table 6).

**Table 6:** PAS Diagnostic Accuracy Statistics Sensitivity, specificity, positive predictive value (PPV), and negative predictive value (NPV) of the Paediatric Appendicitis Score (PAS), with 95% confidence intervals (CI), derived from the 2×2 table in Table 5.​

Statistic​	Value (%)​	95% CI​
Sensitivity​	96.6%​	93.4-98.3%​
Specificity​	12.5%​	6.5-22.8%​
PPV​	80.1%​	75.1-84.4%​
NPV​	50.0%​	28.0-72.0%​

Receiver operating characteristic curve analysis of PAS against histopathological outcome, using 298 patients, yielded an AUC of 0.730 (95% CI 0.663-0.798) (Figure 3).

**Figure 3:**
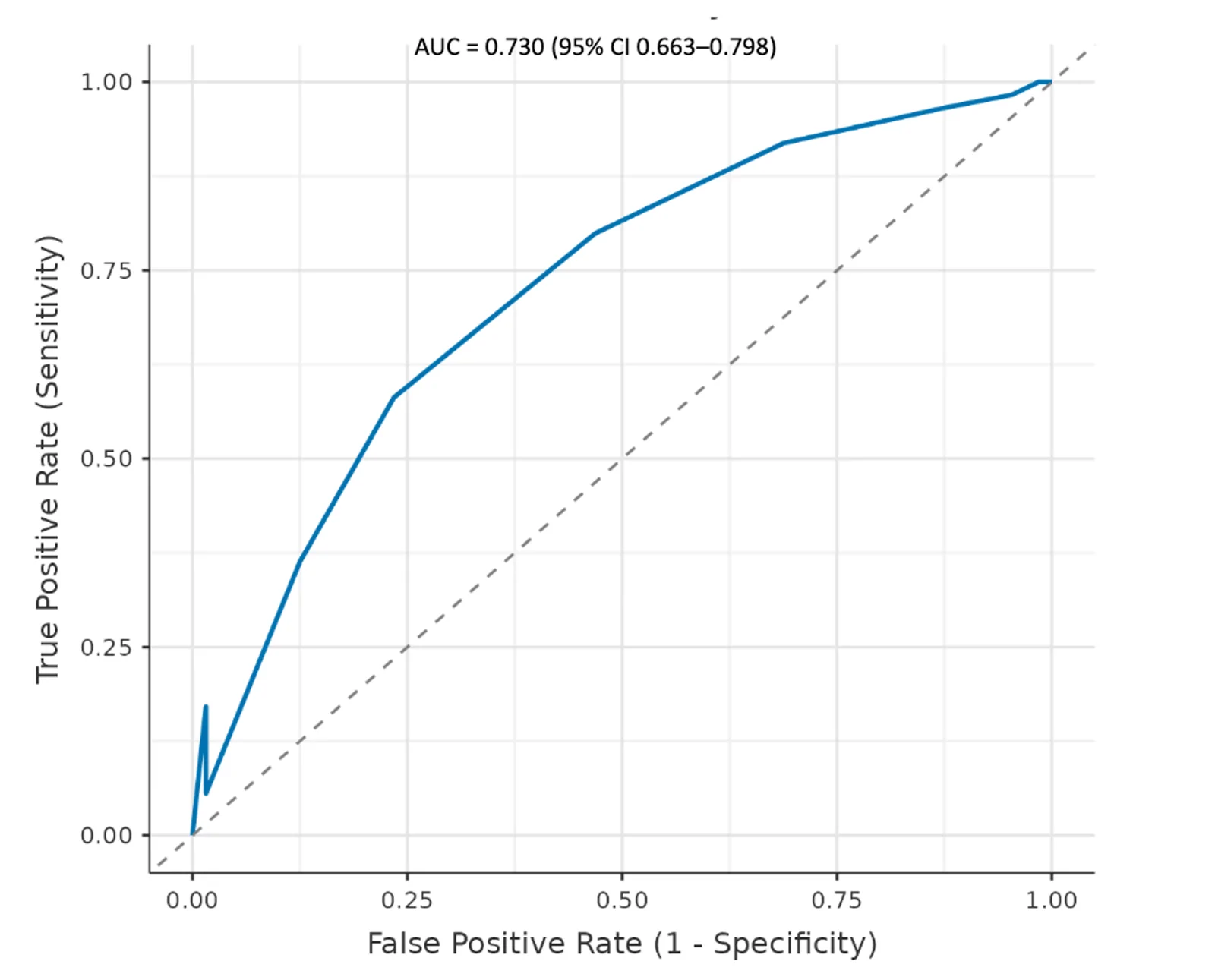
ROC Curve for PAS in Predicting Appendicitis​ Receiver operating characteristic (ROC) curve for the Paediatric Appendicitis Score (PAS) against histopathological outcome (n=298). Area under the curve (AUC) = 0.730 (95% confidence interval (CI) 0.663-0.798), indicating fair discriminative ability.​

NAR by PAS risk category was also analysed. Low risk had an NAR of 50.0% (8/16 patients), equivocal risk of 31.1% (41/132 patients), and high risk of 10.0% (15/150 patients). A chi-square test confirmed this association was statistically significant (χ²=26.63, p ≈ 1.65 × 10⁻⁶) (Figure 4). 

**Figure 4:**
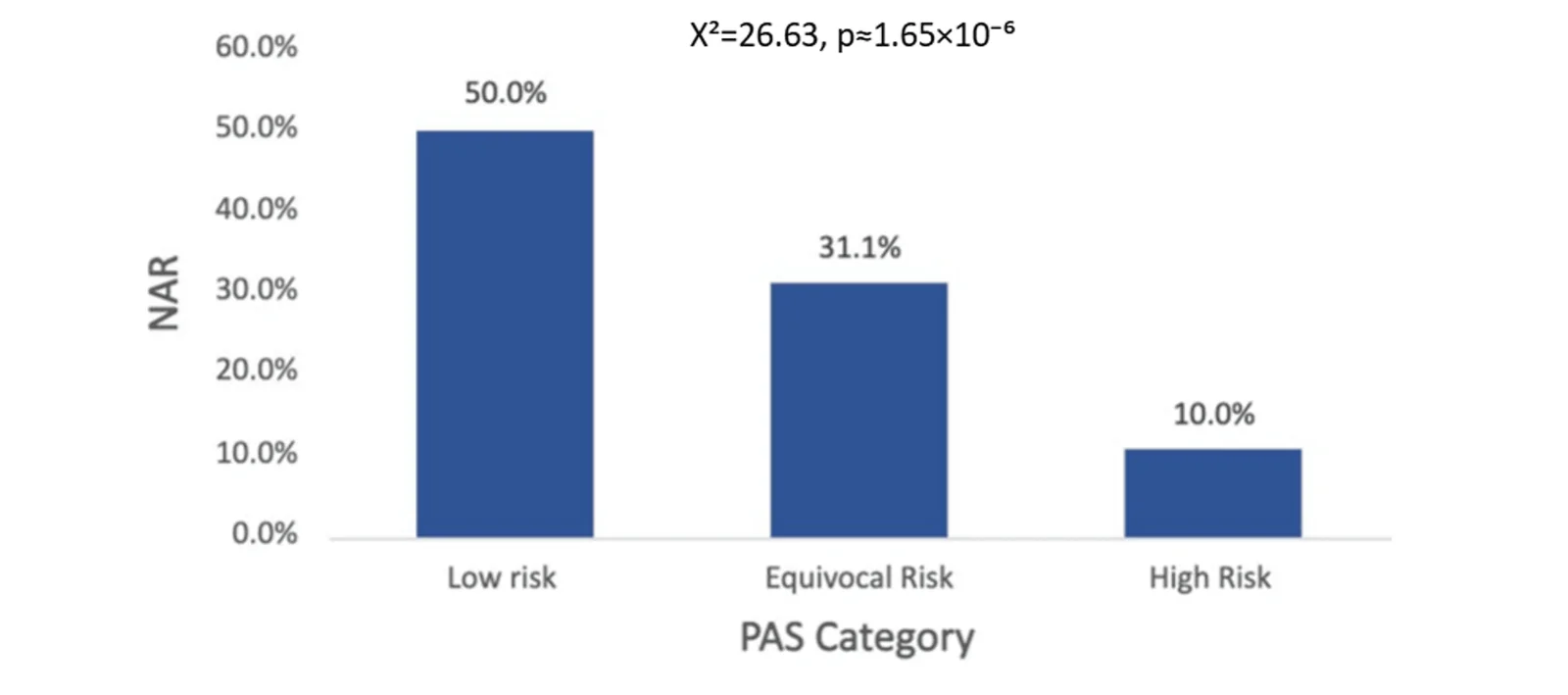
NAR by PAS Risk Category​ Negative appendicectomy rate (NAR) stratified by Paediatric Appendicitis Score (PAS) risk category (n=298). NAR declined progressively from low risk (50.0%) to equivocal risk (31.1%) to high risk (10.0%), a statistically significant association (χ²=26.63, p≈1.65×10⁻⁶).

Of the 318 explored patients, 108 (34.0%) underwent imaging: 103 had ultrasound, 3 had CT, 1 had MRI, and 1 had abdominal X-ray. Imaging use was also reported by PAS risk category among the 314 operated patients with a documented PAS risk category (318 operated patients, excluding the 4 with insufficient documentation to calculate PAS); unlike the diagnostic accuracy analyses, this denominator does not require definitive histopathology. Low risk had an imaging rate of 52.6%, equivocal risk of 37.1%, and high risk of 27.6%. A chi-square test confirmed this association was statistically significant (χ²=6.29, p ≈ 0.043) (Figure 5). 

**Figure 5:**
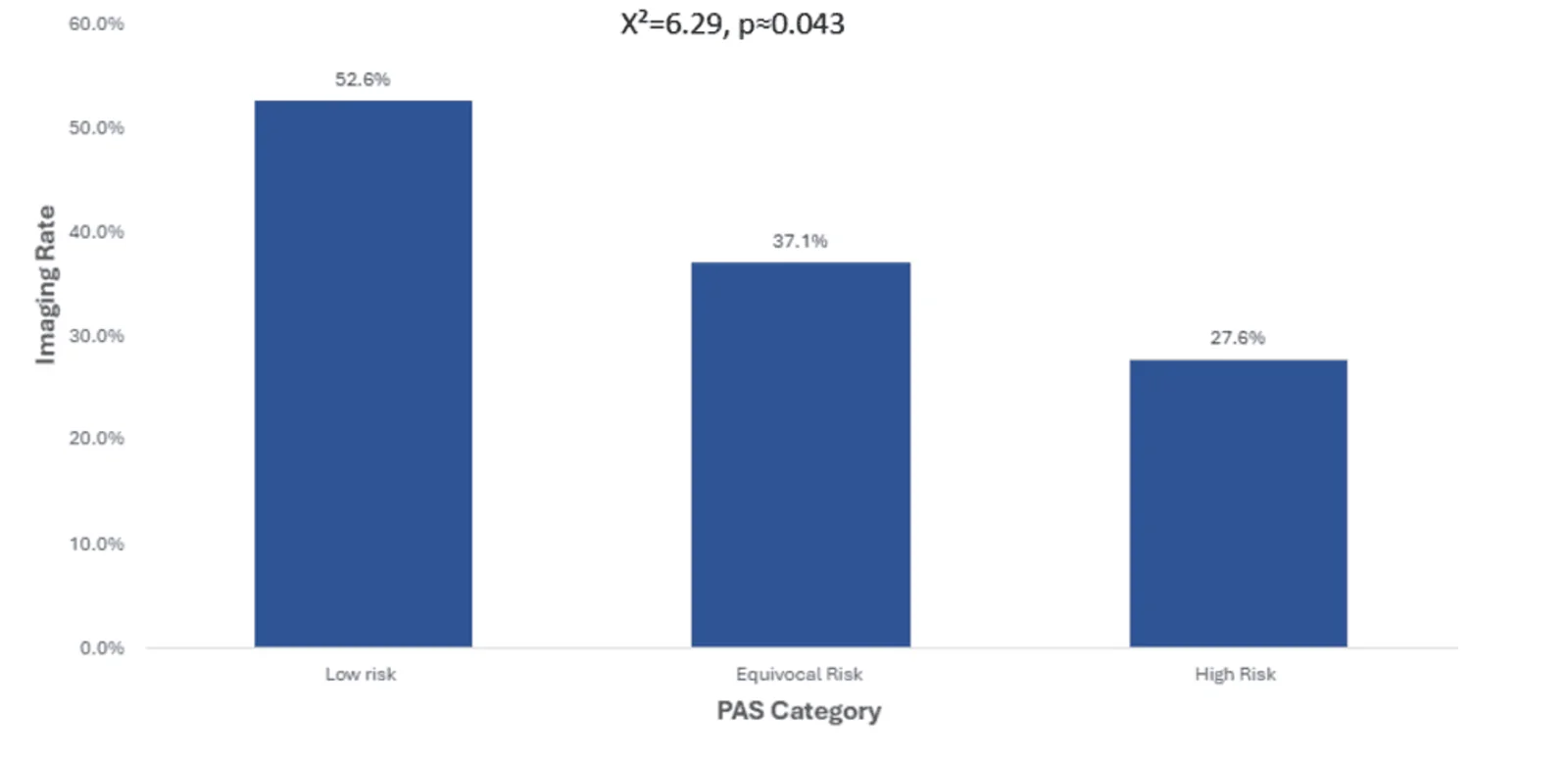
Imaging Use by PAS Risk Category Proportion of patients undergoing imaging, stratified by Paediatric Appendicitis Score (PAS) risk category (n=314). Imaging use declined from low risk (52.6%) to equivocal risk (37.1%) to high risk (27.6%), a statistically significant association (χ²=6.29, p≈0.043).

Additionally, patients categorised as equivocal risk had an NAR of 48.9% (23/47, 95% CI 35.3-62.8%) when imaged compared to an NAR of 21.2% (18/85, 95% CI 13.8-31.0%) when not imaged (Figure 6, Table 7). Fisher's exact test revealed p = 0.0015 and an odds ratio of 3.57 (95% CI 1.65-7.73).

**Figure 6:**
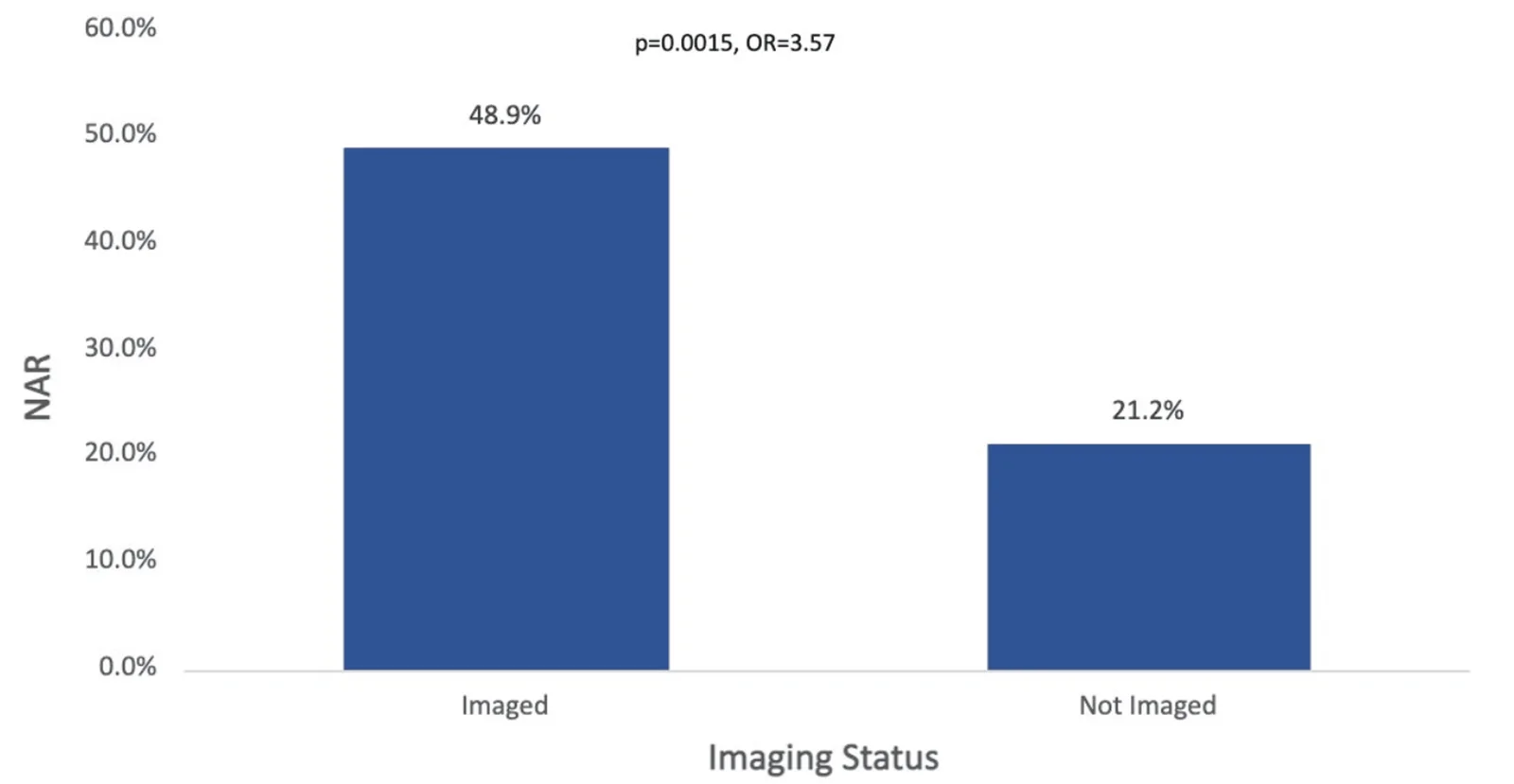
NAR by Imaging Status Within Equivocal Risk Group​ Negative appendicectomy rate (NAR) among equivocal risk patients, stratified by imaging status. NAR was significantly higher among imaged patients (48.9%) than those not imaged (21.2%) (Fisher's exact test, p = 0.0015, odds ratio (OR) = 3.57).​

**Table 7:** PAS Category, Imaging, and Histological Outcome Cross-Tabulation​ Cross-tabulation of Paediatric Appendicitis Score (PAS) risk category, imaging status, imaging conclusion, and histopathological outcome among operated patients with a documented PAS (n=314). Imaging conclusion: Positive = appendix visualised with evidence of inflammation; Negative = appendix visualised without inflammation; Inconclusive = appendix not visualised. "Missing"= no definitive histopathology (e.g. appendix left in situ). NAR (negative appendicectomy rate) = normal appendix / (normal appendix + appendicitis), so it excludes patients with missing histology. This reconciles the 53 imaged equivocal-risk patients with the denominators underlying the reported NARs of 48.9% (23/47 with available histology) and 21.2% (18/85 with available histology). Four further operated patients (three imaged, one not imaged) had insufficient documentation to calculate a PAS and are not shown; including them, the full operated cohort totals 318.​

PAS Category​	Imaging status​	Imaging conclusion​	Normal appendix​	Appendicitis​	Missing/Indeterminate​	Total, n​	NAR n/N (%)​
​ ​ ​ Low risk ​	​ ​ Imaged​	Positive​	0​	3​	1​	4​	0/3 (0.0%)​
		Negative​	2​	0​	0​	2​	2/2 (100.0%)​
		Inconclusive​	1​	2​	1​	4​	1/3 (33.3%)​
		Subtotal​	3​	5​	2​	10​	3/8 (37.5%)​
	Not Imaged​	Not applicable​	5​	3​	1​	9​	5/8 (62.5%)​
​ ​ ​ Equivocal risk ​	​ ​ Imaged​	Positive​	4​	10​	4​	18​	4/14 (28.6%)​
		Negative​	1​	3​	0​	4​	1/4 (25.0%)​
		Inconclusive​	18​	11​	2​	31​	18/29 (62.1%)​
		Subtotal​	23​	24​	6​	53​	23/47 (48.9%)​
	Not Imaged ​	Not applicable ​	18​	67​	5​	90​	18/85 (21.2%)​
​ ​ ​ High risk ​	​ ​ Imaged​	Positive​	2​	16​	0​	18​	2/18 (11.1%)​
		Negative​	2​	0​	1​	3​	2/2 (100.0%)​
		Inconclusive​	6​	15​	0​	21​	6/21 (28.6%)​
		Subtotal​	10​	31​	1​	42​	10/41 (24.4%)​
	Not Imaged​	Not applicable​	5​	104​	1​	110​	5/109 (4.6%)​

A similar pattern was observed within the triple-screen-negative subgroup: NAR was 81.5% (22/27, 95% CI 63.3-91.8%) among imaged patients compared with 63.6% (14/22, 95% CI 43.0-80.3%) among those not imaged (Figure 7). Fisher's exact test revealed p = 0.202 and an odds ratio of 2.51 (95% CI 0.68-9.25). Neither association should be interpreted as evidence that imaging causes unnecessary surgery; both more plausibly reflect confounding by indication, whereby imaging may have been preferentially requested in more diagnostically uncertain presentations. 

**Figure 7:**
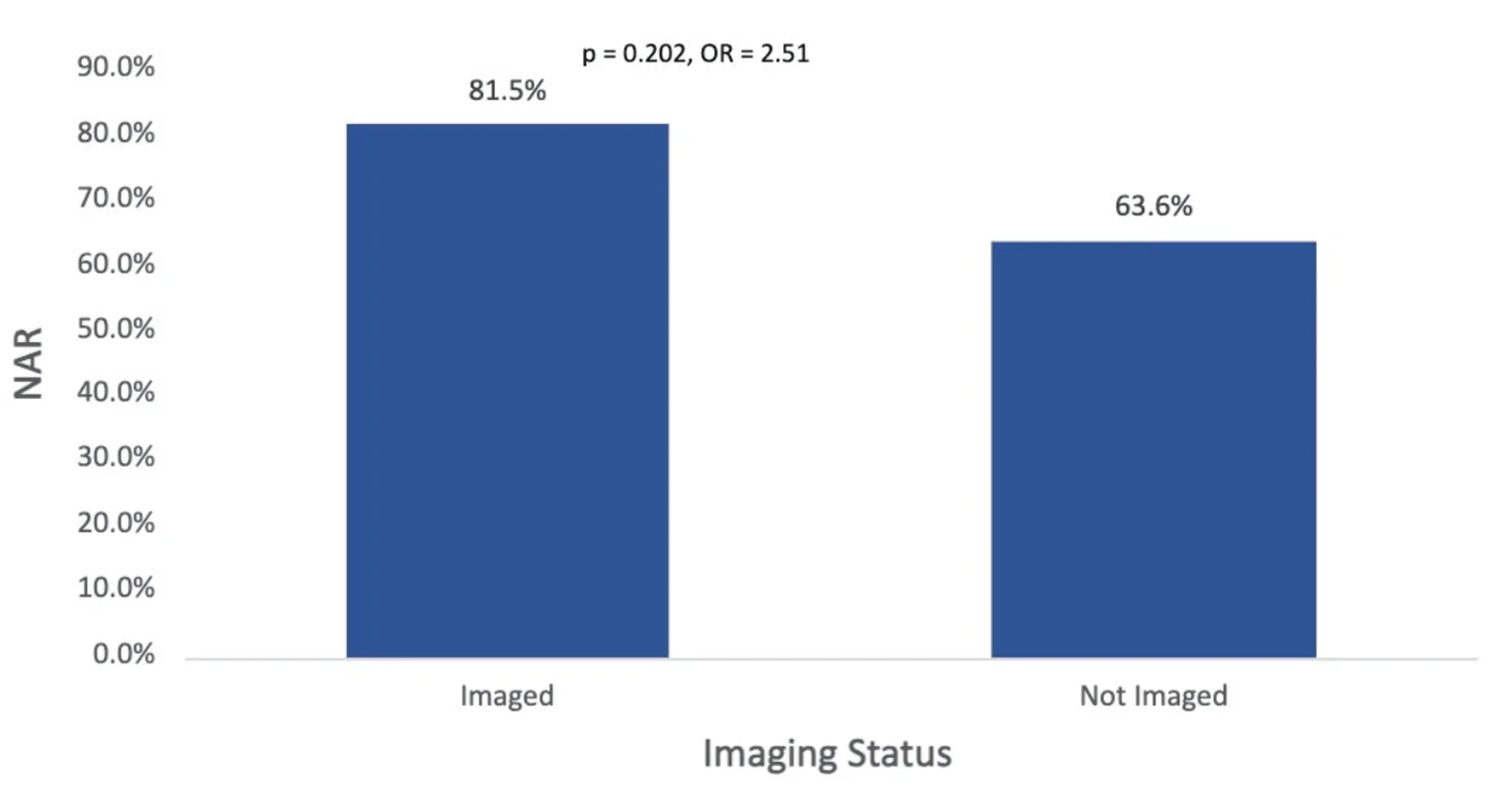
NAR by Imaging Status Within Triple-Screen-Negative Subgroup​ Negative appendicectomy rate (NAR) among triple-screen-negative patients, stratified by imaging status. NAR was numerically higher among imaged patients (81.5%) than those not imaged (63.6%), though this difference did not reach statistical significance (Fisher's exact test, p=0.202, odds ratio (OR) =2.51).​

A further subgroup of 11 patients met both AoMRC triple-screen-negative criteria and had a low-risk PAS score yet proceeded to surgery. Of the 9 with histology, 6 (66.7%, 95% CI 35.4-87.9%) had a normal appendix, and 3 (33.3%, 95% CI 12.1-64.6%) had appendicitis, including one complicated case. The wide intervals reflect the very small subgroup, and this analysis is exploratory. 

## Discussion

Key clinical summary 

The key finding of this audit was an NAR of 21.2%, which significantly exceeded the GIRFT-reported national NAR figure of 10% (p = 7.54 × 10⁻⁹) [6]. Additionally, over half of all the negative appendicectomies in the audit (56.3%) occurred in patients who had already met the AoMRC’s triple-screen-negative criteria before surgery. Exploratory analysis suggested that PAS carried diagnostic value despite not being used prospectively during the study period, and that imaging use - which was not systematically protocolised - varied in a way that did not consistently track diagnostic uncertainty. 

Negative appendicectomy rate 

The histopathology NAR in the present study was 21.2%, significantly higher than the GIRFT-reported national NAR figure of 10% (p < 0.0001) [6]. Published paediatric literature reports considerable variation in NAR, ranging from 15.9% in the 139-centre multicentre RIFT study [17] to 48% within the paediatric subgroup of a single-centre retrospective cohort study [18]. A recent UK teaching hospital audit reported a near-identical negative appendicectomy rate of 23.6% within their paediatric cohort, alongside limited use of validated clinical scoring systems (16.7%) [19]. Although the specific factors underlying RIFT's lower rate are not reported, a Dutch national audit had a negative appendicectomy rate of 3.1% in a cohort where pre-operative imaging was used in 98.9% of children and concluded that routine imaging was likely the driver of the low NAR [20]. This is consistent with the present audit's findings that imaging use was inconsistent (34.0% of the operated cohort) and was not associated with a lower NAR. 

This elevated rate carries clinical consequence: complications requiring hospital readmission occurred in 5 of the 64 negative appendicectomies (7.8%), highlighting that removal of a histologically normal appendix is not without morbidity. Potential contributing factors specific to this cohort, including the use of biochemical screening criteria and a validated clinical scoring system, are considered in the following sections.

AoMRC triple-screen-negative criteria 

AoMRC guidance states explicitly that imaging is not recommended when the triple screen is negative, given its reported NPV >99% [5]; as noted in the Introduction, this guidance applies to adults and children, but the NPV figure's evidentiary basis within the guidance itself appears adult-derived. A comparably high NPV has, however, been independently reported in a paediatric cohort using the same three markers [3], lending some support to its extrapolation to children, albeit without confirmed identical cut-offs. Nonetheless, as the wider AoMRC intervention concerns appendicectomy performed without diagnostic confirmation, we treated a patient's progression to surgery despite a negative triple screen as relevant to this standard - an extension of the guidance's intent, rather than something it states directly. Similar findings have also been reported in a prospective cohort, where a combination of normal white blood cell count (WBC), neutrophil percentage, and CRP achieved a NPV of 100%, based on 63 patients testing negative, none of whom had appendicitis [21], although the CRP cut-off used differed from the present study (<5 mg/L vs <10 mg/L).

In our study, 31/55 (56.4%, 95% CI 43.3-68.6%) of patients who had met the triple-screen-negative criteria underwent imaging. Among the 27 imaged triple-screen-negative patients with histology, 22 (81.5%, 95% CI 63.3-91.8%) had a negative appendicectomy. 

The findings in the audit suggest that ultrasound frequently failed to resolve diagnostic uncertainty in this subgroup, and that other factors such as clinical presentation, history, and examination may have contributed to the decision to proceed to surgery, rather than imaging or biochemical results. On the other hand, 13 patients (26.5%) did have histologically confirmed appendicitis, including one patient with complicated appendicitis. This indicates that the triple-screen should not be used as a definitive basis for ruling out the need for surgery without further clinical correlation. Beyond conventional inflammatory markers, albumin-based composite indices have been investigated in acute care, including appendicitis. Their potential value requires indication-specific validation, and evidence from broader acute and critical care populations should not be extrapolated directly to paediatric rule-out decisions [22]. 

Overall, a substantial proportion of children with a negative triple screen proceeded to surgery, and most of those with histology had a normal appendix (36/49, 73.5%); inconclusive imaging did not resolve the diagnostic uncertainty it was intended to resolve. Whether this reflects persistent clinical concern, evolving findings, alternative diagnoses, or inconsistent application of the triple-screen guidance cannot be determined from this audit. 

Secondary/exploratory analysis: clinical scoring system and imaging use 

In our study, the PAS diagnostic performance was evaluated against histopathology outcomes. Analysis showed sensitivity was 96.6% (95% CI 93.4-98.3%) and specificity was 12.5% (95% CI 6.5-22.8%). This aligns with several trends within published papers, one of which reports that PAS detected 96% (95% CI 91%-98%) of histology-confirmed cases but achieved limited specificity of 31% [23], using the same cut-off PAS categories used for this audit (PAS ≥ 4). Sensitivity at the low-risk cut-off in the present cohort (96.6%, 95% CI 93.4-98.3%) closely matched that reported in a prospective validation study using a closely comparable, though not identical, cut-off (PAS ≤ 4 vs PAS < 4 in the present study) (97.6%, 95% CI 91.6-99.3%) [24]. However, NPV differed substantially (50.0%, 95% CI 28.0-72.0% vs 97.7%, 95% CI 92.0-99.4%), with non-overlapping confidence intervals. The lower NPV likely reflects selection of patients who proceeded to surgery and the high prevalence of appendicitis in this cohort, while the small low-risk subgroup increases uncertainty around the estimate. The calculated AUC in the study was 0.730 (95% CI 0.663-0.798), indicating fair discriminative ability, and closely matching the AUC of 0.73 reported in a comparable UK paediatric cohort [23]. Higher AUCs are also reported in some individual prospective validation cohorts (0.895-0.95) [15,24]. 

NAR also decreased across PAS risk groups, from 50.0% in low-risk to 10.0% in high-risk patients (χ² p ≈ 1.65×10⁻⁶), evidence of risk stratification despite the score's limited specificity at any single cut-off. Although the PAS scores were calculated retrospectively, the results show a clear gradient, supporting prospective evaluation of PAS within the clinical pathway. 

Our study also revealed that imaging use declined as risk category rises, from 52.6% in low-risk to 27.6% in high-risk patients (χ² p ≈ 0.043). This pattern is inconsistent with what might be expected, given that imaging use would be anticipated to be highest in the equivocal-risk category, where diagnostic uncertainty is greatest. Furthermore, within the equivocal-risk group specifically, where imaging might be expected to add the greatest diagnostic value, negative appendicectomy rate was significantly higher among imaged patients than those who were not imaged (48.9%, 23/47, 95% CI 35.3-62.8% vs 21.2%, 18/85, 95% CI 13.8-31.0%; Fisher's exact p = 0.0015, OR = 3.57, 95% CI 1.65-7.73). Of the 53 imaged equivocal-risk patients, 47 had definitive histopathology and contributed to the 48.9% NAR above; the remaining 6 lacked histopathology and were excluded from this calculation. Correspondingly, of the 90 non-imaged equivocal-risk patients, 85 had definitive histopathology (21.2% NAR), and 5 lacked histopathology (Table 7). Among equivocal-risk patients who underwent imaging, the majority (58.5%, 31/53) had an inconclusive result; of the 29 with definitive histopathology, 18 (62.1%) subsequently had a normal appendix, suggesting that inconclusive imaging accounts for a substantial proportion of this pattern. The 11 children who met both triple-screen-negative criteria and had a low-risk PAS illustrate this pattern (see Results). 

Taken together, these findings suggest that diagnostic tools that may be of value in this population were not systematically embedded in the clinical pathway during the study period; whether their more consistent use would reduce unnecessary appendicectomy requires prospective evaluation. 

Synthesis 

Taken together, the findings suggest several possible contributors to the elevated NAR observed in this cohort, although the audit cannot establish a cause. NAR (21.2%) was significantly higher than the GIRFT national figure of 10% (p < 0.0001), and over half of the negative appendicectomies occurred in patients who had already met the triple-screen-negative criteria before surgery (see Table 8 for a summary of both standards).

**Table 8:** Standards Summary Table​ Summary of findings against the two audit standards: the negative appendicectomy rate (NAR) reported nationally by Getting It Right First Time (GIRFT), and the triple-screen guidance from the Academy of Medical Royal Colleges (AoMRC).​

Standard ​	Result​	Interpretation​
GIRFT national NAR figure (10%) [6]​	NAR = 21.2% (p<0.0001)​	Significantly exceeds reported figure​
AoMRC's triple screen (imaging not recommended if negative) [5]​	56.4% imaged despite negative screen; 73.5% NAR within subgroup​	More than half of triple-screen-negative patients underwent imaging despite guidance; whether this reflects inconsistent guidance application, persistent clinical concern, or evolving findings cannot be determined from this audit​.

In exploratory analyses, the Paediatric Appendicitis Score showed fair discriminative ability when applied retrospectively, although it was not used prospectively to guide surgical decisions. Imaging, when obtained, was not associated with a lower NAR and was used inconsistently across risk categories, although confounding by indication is likely. 

The GIRFT acute paediatric abdominal pain and appendicectomy pathway explicitly incorporates clinical risk scoring, inflammatory markers (full blood count and CRP), and ultrasound imaging as sequential steps in the diagnostic assessment of suspected appendicitis, while also acknowledging that ultrasound is not fully sensitive [7]. The findings of this audit are consistent with the elevated NAR reflecting variation in how existing diagnostic tools were used, rather than their absence; however, as adherence and the reasons behind individual surgical decisions were not directly measured, this remains a hypothesis generated by the audit rather than a demonstrated explanation. It suggests that prospective evaluation of more consistent, protocolised use of PAS, inflammatory markers, and imaging together - rather than development of new diagnostic tools - is a reasonable next step for reducing unnecessary appendicectomy in this unit. 

Limitations 

Our study represents a single-centre retrospective analysis, which may limit external validity and the generalisability of findings to other institutions or populations. This audit spanned an 8.5-year period; however, clinical practice, staffing, and diagnostic pathways may have evolved over this time, and findings should be interpreted as a pooled assessment across this period rather than a snapshot of current practice alone. Because cases were identified through manual review of ward and team admissions rather than a formal diagnostic or procedure coding search, ascertainment depended on accurate ward allocation and admission documentation at the time; a coded search strategy might have identified a different case set, and the two approaches cannot be directly compared in this audit. 

The GIRFT national NAR benchmark used for comparison reflects data collected in 2017-2018, predating most of the present 8.5-year audit period, and a fixed-benchmark binomial comparison cannot account for sampling uncertainty in this reported national rate or for case-mix differences between centres. PAS was also calculated retrospectively, relying on the accuracy and completeness of existing electronic patient records; although data were independently extracted by two reviewers to minimise inter-observer variability, some potential for documentation bias remains. As an inherent limitation of retrospective symptom scoring, a PAS component recorded as absent may reflect either a true negative finding or an undocumented one; triangulating Emergency Department, surgical, and nursing triage documentation reduces but does not eliminate this risk. The underlying documentation was not retained in a form that distinguishes explicitly negative from undocumented findings, precluding a sensitivity analysis restricted to fully documented cases. 

Sixteen children whose macroscopically normal appendix was left in situ had no histopathology and were excluded from the NAR denominator, as the rate describes appendicectomies. Counting these as negative explorations would give 80/318 (25.2%) negative surgical explorations, so the reported NAR may understate the proportion of children explored without appendicitis. Diagnostic accuracy findings, including PAS sensitivity, specificity and AUC, are derived exclusively from the operated cohort with histopathology (the reference standard), which introduces verification bias and limits generalisability to all children presenting with suspected appendicitis. Negative appendicectomy was defined using standard histopathological criteria alone, as inconsistent reporting of inflammatory grading and microbiological findings precluded a stricter definition [8]. 

As a retrospective study, this audit could not control patient selection, diagnostic decision-making, or intraoperative management. The decision to operate rested largely on individual surgeon judgement rather than a standardised protocol, introducing variability in diagnostic thresholds inherent to retrospective designs. The observed associations between imaging use and negative appendicectomy rate are correlational; as a retrospective, non-randomised study, this audit cannot establish causation, and residual confounding - particularly confounding by indication, whereby imaging was more likely to be requested in diagnostically uncertain presentations - cannot be excluded. The timing of inflammatory marker sampling relative to symptom onset, imaging, and surgery was not systematically recorded, precluding assessment of whether marker trends over time influenced triple-screen classification. 

Future studies should aim to address these limitations by introducing a prospective, multi-centred, and standardised research design that captures contemporary diagnostic practice. Prospective studies that implement the use of clinical scoring, interpretation of inflammatory markers, and imaging use, similar to the recommended GIRFT paediatric acute abdominal pain and appendicectomy pathway [7], with measurement of post-operative outcome follow-up, would aim to improve data reliability and produce more comprehensive data on the negative appendicectomy rate. 

Recommendations for practice 

This audit found a negative appendicectomy rate of 21.2%, higher than the national average reported in the GIRFT specialty report [6]; the following recommendations are based on these findings and comparable paediatric literature. 

Prospective documentation of a validated clinical score (such as PAS) at initial assessment, built into the EPR, should be considered. The negative appendicectomy rate fell progressively across PAS risk categories, from 50.0% in low-risk to 10.0% in high-risk patients (χ² p ≈ 1.65×10⁻⁶), and PAS demonstrated fair discriminative ability overall (AUC 0.730, 95% CI 0.663-0.798) - despite having been calculated retrospectively and never used to guide clinical decision-making in this cohort.

A defined local pathway should be considered for children meeting the AoMRC triple-screen-negative criteria, given that 36 of 49 (73.5%) with histology had a normal appendix. Furthermore, although the subgroup was small (n=11), the combination of biochemical inflammatory markers and clinical scoring together may provide further diagnostic value, with 6 of 9 with histology found to have a normal appendix. 

Local imaging pathway posters, providing clearer escalation guidance for imaging in equivocal-risk patients, should be considered. Imaging use declined across PAS risk categories, from 52.6% in low-risk to 27.6% in high-risk patients (χ² p ≈ 0.043). Imaging use was not highest in the equivocal-risk group (37.1%), where diagnostic uncertainty - and therefore the potential value of imaging - should arguably be greatest. 

The GIRFT acute abdominal pain and appendicectomy pathway represents an important clinical tool for standardising the diagnosis and management of suspected appendicitis in children. Since the GIRFT programme first highlighted variation in paediatric abdominal pain care across NHS trusts, the national negative appendicectomy rate has almost halved, from 11.5% to 5.8% [25], though this trend is reported in a GIRFT improvement-programme update rather than a formally audited national report, and should be interpreted as an indicative improvement trend rather than a directly comparable benchmark. 

A future re-audit over a shorter, defined cycle following implementation of these recommendations would allow more direct assessment of current practice and completion of the audit cycle. This audit did not include an intervention; these recommendations represent a proposed future quality improvement project, not a tested change.

## Conclusions

This audit found a negative appendicectomy rate of 21.2%, significantly higher than the GIRFT-reported national figure (p < 0.0001), though within the range reported in the wider paediatric literature. Over half of the negative appendicectomies occurred in children who met the AoMRC triple-screen-negative criteria. While appendicitis in children can present atypically, these findings identify an opportunity to review how biochemical screening, clinical scoring and imaging are used within the local diagnostic pathway. Prospective evaluation is required to determine whether standardised, combined use of these tools can reduce unnecessary appendicectomy without increasing missed or delayed diagnoses. 
